# Effects of Titanium Dioxide Nanoparticles on Chick Embryo: Immunomodulatory, Hepatic and Biochemical Alterations

**DOI:** 10.1002/vms3.70105

**Published:** 2024-10-30

**Authors:** Md. Sadequl Islam, Md. Nurul Amin, Mst. Deloara Begum

**Affiliations:** ^1^ Department of Anatomy and Histology Faculty of Veterinary and Animal Science Hajee Mohammad Danesh Science and Technology University Dinajpur Bangladesh; ^2^ Department of Animal Science and Nutrition Faculty of Veterinary and Animal Science Hajee Mohammad Danesh Science and Technology University Dinajpur Bangladesh; ^3^ Department of Microbiology Faculty of Veterinary and Animal Science Hajee Mohammad Danesh Science and Technology University Dinajpur Bangladesh

**Keywords:** biochemical changes, chick embryo, hepatic alterations, immunomodulatory effects, nanoparticles, titanium dioxide

## Abstract

**Background:**

The utilization of titanium dioxide nanoparticles (TiO_2_ NPs) has significantly increased across various industries.

**Objectives:**

This study rigorously explored the impact of TiO_2_ NPs exposure on chicken embryos, focusing particularly on alterations in the immune system, liver functionality and key biochemical markers.

**Methods:**

The study involved three groups of 30 eggs each, subjected to increasing doses of TiO_2_ NPs: Group C (control), Group T1 (150 µg/mL) and Group T2 (300 µg/mL). After 48 h of incubation, the eggs in Groups T1 and T2 each received an injection of 0.3 mL of the TiO_2_ NPs solution. In contrast, the eggs in the control group (Group C) were injected with 0.3 mL of saline solution. Histopathological changes were analysed using haematoxylin and eosin (H&E) staining, whereas amniotic fluid's biochemical properties were examined photometrically. The study also assessed the expression of immune genes (*AvBD9, IL6* and *IL8L2*) through quantitative PCR. The evaluations included growth metrics, amniotic fluid biochemistry and histological analysis of the liver, caecal tonsil and bursa of Fabricius.

**Results:**

The results revealed subcutaneous haemorrhage, significant reductions in total body weight and marked changes in biochemical markers, including urea, creatinine, alkaline phosphatase (ALP), aspartate aminotransferase (AST) and alanine aminotransferase (ALT), in the amniotic fluid of the groups treated with TiO_2_ NPs, compared to the control. Histological examinations indicated noticeable alterations in the liver, caecal tonsil and bursa of Fabricius following TiO_2_ NP exposure. These alterations were characterized by disruptions in cellular structures and variations in lymphocyte counts. Furthermore, a notable decrease in the expression of immunity genes, namely, *AvBD9, IL8L2* and *IL6*, was observed in the TiO_2_ NP‐treated groups compared to the control.

**Conclusion:**

The findings underscore the need for risk assessments of TiO_2_ NPs exposure due to its impact on development and immunity. Future research should explore its impact on neurodevelopment and degeneration.

## Graphical abstract

Exposure to titanium dioxide nanoparticles (TiO_2_ NPs) in chick embryos caused subcutaneous haemorrhage, reduced body weight and elevated biochemical markers (urea, creatinine, ALP, AST and ALT) in amniotic fluid. Histological changes in the liver, caecal tonsil and bursa of Fabricius were observed, including cellular disruptions and altered lymphocyte counts. TiO_2_ NP‐treated groups also showed reduced expression of immune genes (*AvBD9, IL8L2* and *IL6*), indicating significant immunomodulatory and hepatic effects of TiO_2_ NP exposure.

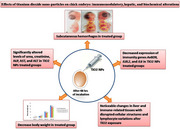

## 1 Introduction

The rapid advancement of nanotechnology has led to groundbreaking developments across various sectors, including manufacturing, textiles, communication and healthcare, as highlighted by Oberdorster, Oberdörster, and Oberdörster (2005); Nel et al. (2006) and Malik, Muhammad, and Waheed (2023). This technological surge has profoundly transformed consumer products, evidenced by the integration of nanotechnology in over 1800 commercial items (Vance et al. 2015). Titanium dioxide nanoparticles (TiO_2_ NPs) play a pivotal role in this revolution, finding applications in pigments, paints, polymers and food products. However, the escalating utilization of TiO_2_ NPs has raised considerable concerns regarding its potential impacts on human health and environmental safety. Therefore, it is imperative to conduct a thorough assessment of the advantages and risks associated with TiO_2_ NPs to understand their overall implications (Dalai et al. 2013). Anticipated to reach a global production capacity of 2.5 million metric tons by 2025, TiO_2_ NPs have become integral in numerous industries, attributable to their multifunctional capabilities and distinctive properties. The photocatalytic activity and characteristics of TiO_2_ NPs, especially when the particle size is below 100 nm, set them apart from their larger counterparts, thus making them invaluable in a wide array of industrial applications and consumer products (Lehner et al. 2013; Rashid, Forte Tavcer, and Tomsis 2021). The employment of nano‐sized TiO_2_ enhances stability, anti‐corrosion characteristics and photocatalytic efficiency. These improvements have paved the way for innovative applications in diverse fields such as agriculture, medicine and construction, offering new frontiers for technological advancements (FDA 2002).

TiO_2_ NPs are extensively utilized in a variety of consumer products, including sunscreens, paints and plastics, underscoring their crucial role in modern technological advancements and consumer well‐being (Wakefield, Stott, and Hock 2005; Guarino, Costa, and Porro 2008). However, recent studies have shed light on the potential adverse effects associated with exposure to nano‐titanium dioxide. Hashem et al. (2020) reported that exposure to titanium dioxide leads to dose‐dependent alterations in various haematological parameters and immune responses in rats. This includes modifications in leukocyte profiles, immunoglobulin levels and lymphocyte activity, thus indicating a multifaceted impact on the immune system. Lappas (2015) found that titanium dioxide can be internalized by immune cells, potentially resulting in immunomodulatory and immunotoxic effects. TiO_2_ NPs are considered hazardous due to their capacity to generate reactive oxygen species (ROS), which can inflict cellular damage, provoke inflammatory responses and modify gene expression (Gojznikar et al. 2022). These findings raise substantial concerns regarding the overall impact of TiO_2_ NPs on human health and safety (Ma et al. 2009; Lafuente et al. 2003). Previous animal studies have consistently demonstrated that nano‐TiO_2_ particles, upon administration, tend to disperse widely throughout the body. They exhibit significant penetration of cell membranes, accumulation in mitochondria and deposition in various vital organs. Their presence has been linked to a variety of organ dysfunctions, notably affecting the lungs, liver, kidneys and brain, as evidenced by a range of research findings (Liu et al. 2009; Halappanavar et al. 2011; Cui et al. 2012; Gui et al. 2011; Ze et al. 2014). Furthermore, laboratory experiments have confirmed the deleterious effects of nano‐TiO_2_, demonstrating its propensity to induce oxidative stress, inflammation, DNA damage, apoptosis, genotoxicity and alterations in enzyme functionality (Minghui et al. 2023). According to Park et al. (2008) and Zhao et al. (2009), the cellular responses to nano‐TiO_2_ can manifest as programmed cell death or tissue necrosis. More recent research (Minghui et al. 2023; Ali et al. 2019) has further elucidated the detrimental effects of nano‐TiO_2_ exposure in animal models. Minghui et al. (2023) highlighted the adverse impact of nano‐TiO_2_ on mammalian reproduction, noting its accumulation in reproductive organs and negative effects on the development of gametes and offspring, mediated through mechanisms like oxidative stress and hormonal disruption. Ali et al. (2019) reported significant biochemical, genetic and histological disruptions in the liver of mice exposed to TiO_2_ NPs, with these effects being more pronounced in smaller particle sizes and at higher doses. Remarkably, exposure to TiO_2_ NPs has been observed to cause dysfunction in pulmonary macrophages and trigger splenocyte death (Baranowska‐Wójcik et al. 2020). Moreover, the extensive body of scientific literature has well documented the accumulation of ROS, changes in cytokine production and compromised immune function due to TiO_2_ NPs (Li et al. 2008; Liu et al. 2011; Sang et al. 2012). Despite this wealth of information, the immunotoxic effects of nano‐TiO_2_ remain insufficiently explored. The present study addresses this gap by adopting a novel approach, focusing on nano‐TiO_2_’s impacts during the vital early developmental stages of the embryo, a period characterized by intense growth and susceptibility to external influences. The study aims to broaden the current understanding by investigating how nano‐TiO_2_ affects the embryonic microenvironment, specifically assessing biochemical changes in amniotic fluid and the modulation of immune genes. The findings contribute valuable insights to the existing knowledge base. In this context, the present research examines the nuanced effects of TiO_2_ NPs on the chick embryo, a model selected for its physiological similarities to the human embryo and its demonstrated efficacy in developmental toxicity assessments (Patel et al. 2018). The study is designed to deepen our understanding of the potential risks and impacts associated with TiO_2_ NPs exposure during critical embryonic development stages. The present study explores the effects on the immune system, liver function and biochemical processes. Analysing biochemical changes in the amniotic fluid is key to understanding the embryo's microenvironment and the potential repercussions of TiO_2_ NPs exposure. Because the amniotic fluid forms a vital link between the embryo and its environment, its analysis can shed light on how nanoparticles influence developmental processes and immediate physiological responses (Da Da Silva et al. 2019). This knowledge is crucial for assessing the potential hazards and developmental implications of TiO_2_ NPs on embryonic growth. The findings provide significant insights into the fundamental mechanisms and potential risks associated with TiO_2_ NPs, elucidating their impact on embryonic development and broader implications for avian health. By highlighting these critical aspects, the study contributes to informed decision‐making and guides the responsible use of TiO_2_ NPs across various industrial and consumer applications.

## 2 Materials and Methods

### 2.1 Chemicals and Reagents

The TiO_2_ NPs used in our study were procured from Z.h. Scientific and Chemicals Mart, Bangladesh. These particles were characterized by a size smaller than 100 nm and exhibited a high purity level of 99.5%. It is crucial need to understand the environmental exposure and health impacts of TiO_2_ nanoparticles on avian species, particularly considering their potential ingestion through food sources. Weir et al. (2012) established a baseline for TiO_2_ consumption in human dietary intake; however, its direct relevance to avian species necessitates further investigation. In this regard, the estimated exposure of birds to TiO_2_ particles can be deduced on the basis of their diet. The estimated average consumption of TiO_2_ in eggs, ranging from 50 to 100 µg per egg (considering an average egg weight of 52 ± 1.3 g), allows us to evaluate the potential impact on birds when extrapolated to their dietary habits. To fully understand the environmental relevance and potential health risks, it is crucial to assess these exposures in relation to the body weight and dietary variations of different avian species. This approach would allow for a more accurate assessment of the ecological impact of TiO_2_ NPs, particularly in terms of bioaccumulation and the potential effects on avian health and the broader ecosystem. In accordance with the method outlined by Patel et al. (2018), the TiO_2_ NPs were characterized. Subsequently, these nanoparticles were suspended in saline solutions at concentrations of 150 and 300 µg/mL. The suspensions were then subjected to probe sonication for 30 min using an Ultrasonic Cleaner (Model: TUC‐32, China).

### 2.2 Egg Procurement and Dose Administration

Fertile chicken eggs from the Sonali crossbreed (*Gallus gallus domesticus*), with an average weight of 52 ± 1.3 g, were provided by the Government Poultry Farm located in Rangpur‐5400. Prior to incubation, these eggs were stored at 12°C for 2 days. The in ovo experimental procedures were meticulously adhered to, in‐line with the standard operating protocols established in our laboratory (Islam et al. 2023). A total of 90 eggs were used in the study, divided equally into three groups of 30 eggs each. The groups were designated as follows: Group C (control group): This group included 30 eggs that were injected with 0.3 mL of saline solution, serving as the control. Group T1: Comprising 30 eggs, each treated with a dose of 150 µg/mL TiO_2_ NPs. Group T2: Consisting of 30 eggs, each receiving a dose of 300 µg/mL TiO_2_ NPs. Forty‐eight hours post‐incubation, test samples (0.3 mL/egg) were administered into the air sac utilizing sterile 1 mL tuberculin syringes. Following this, the eggs underwent an additional 18‐day incubation period, as detailed by Patel et al. (2018). The incubation environment was precisely controlled using a mechanical system designed to regulate temperature, humidity and forced air ventilation. To ensure optimal development, the eggs were placed in specialized holders and rotated once every 2 h, amounting to a total of 12 rotations daily. In the study, embryo viability was monitored through the daily candling of eggs, performed across the 3 groups of 30 eggs each, which were subjected to varying levels of TiO_2_ NP treatment, including the control group. This daily candling process was an integral part of the experimental protocol, alongside maintaining the incubation conditions at 37.5°C and 60% humidity. The control group (Group C) and the treated groups (Groups T1 and T2) were carefully monitored for temperature fluctuations. Through consistent monitoring and necessary adjustments to ensure egg stability, the incubator's automatic temperature management system successfully maintained a steady temperature of 37.5°C throughout the trial.

### 2.3 Specimen Collection

Five embryo samples were collected on Days 7, 10, 14 and 18 post‐incubation at each respective time point to measure the overall body weight as an indicator of growth. On Day 18, amniotic fluid samples were collected and centrifuged for the analysis of biochemical parameters, which included enzyme activities of alkaline phosphatase (ALP), aspartate aminotransferase (AST) and alanine aminotransferase (ALT), along with urea and creatinine levels. The harvested embryos on each day, after being cleansed with saline, were euthanized through a humane method involving cooling. To examine them, an incision was made at the air sac end of the eggs. We utilized a Radwag Wagi Electroniczne digital weight scale (Model: AS 220.R2, S/N: 544687, range: 0.1 mg–220 g, Made in Poland) to ascertain the total body weights of the subjects. The liver, caecal tonsil and bursa of Fabricius were then carefully collected on Day 18 of incubation. For histological examination, these tissues were preserved in 10% neutral buffered formalin.

### 2.4 Histopathological Studies

After removing formalin from the tissues, they underwent a dehydration process utilizing alcohol in progressively increasing concentrations: 70%, 80%, 90%, 95% and finally 100%. As outlined in the study by Islam et al. (2023), each concentration of alcohol was applied for 1 h to facilitate effective dehydration. Next, using the prescribed method, the tissues were first placed in xylene‐1 for 90 min, followed by another 90 min in xylene‐2. The tissues were then submerged in liquid paraffin at 60°C for 90 min, subsequently cooled and moulded into paraffin blocks. The paraffin blocks were cut into sections measuring 6 µm in thickness with microtome (LEICA RM2125 RTS, made in United States). The sections were then extended by floating them in water at 45°C, before being placed onto clean, oil‐free glass slides and dried in a hot air oven at 62°C for 20 min. Following drying, the slides were stained with haematoxylin and eosin (H&E) and sealed with a coverslip using Canada balsam adhesive, adhering to the protocol recommended by Drury (1983). To accurately capture the results, high‐resolution microphotographs of selected tissues were taken under 10×, 40× and 100× microscopic magnifications using an Amscope (MA500) camera attached to a Richter Optica biological microscope, Model: U‐2T (Carlsbad, California). The quantification of lymphocytes in the caecal tonsils, measurement of hepatocyte diameter and enumeration of uninucleated and binucleated hepatocytes were performed using Image J software (Curvo et al. 2020). In accordance with Islam et al. (2023), a predetermined field length was used for cell counting. For each experimental group, five slides were analysed to determine cell counts.

### 2.5 Evaluation of Biochemical Parameters of Amniotic Fluid

On Day 18 of incubation, the biochemical composition of the amniotic fluid was determined using an 18‐gauge syringe. We collected amniotic fluid samples from five individual eggs in each group. After carefully removing the eggshell's air sac and membranes, the amniotic fluid from each egg was centrifuged at 3000 g for 15 min to separate the supernatant for analysis. Photometry (Siemens Advia 1800, Germany) was used in order to analyse samples of amniotic fluid for the presence of urea, creatinine, ALP, AST and ALT.

### 2.6 Evaluation of Immunity Genes Expression Due to TiO_2_ NPs Exposure

AvBD9, IL8L2 and IL6 expression were assessed by quantitative polymerase chain reaction (qPCR), with ACTB (β‐actin) as the housekeeping gene (Islam et al. 2023). β‐Actin was selected as the reference gene due to its stable expression across samples, and its CT (cycle threshold) values were utilized to normalize gene expression levels. The ΔCT for each sample was calculated by subtracting the CT value of β‐actin from the CT of the gene of interest (GOI), with the formula ΔCT = CT_GOI_  − CT_β‐actin_, to normalize the GOI's expression against the reference gene. Then the average ΔCT value was computed for control samples as a baseline. The ΔΔCT value for each experimental sample was determined by subtracting this average ΔCT of the control group from the sample's ΔCT, following the formula ΔΔCT = ΔCT_Sample_ − ΔCT_βControl (average)._ Finally, the relative gene expression was calculated as 2−^ΔΔCT^, representing the fold change in the expression of the GOI in experimental samples as compared to controls (Livak & schmittgen, 2001). The caecal tonsils of the chick embryo were smashed and homogenized with liquid nitrogen at Day 18 to extract RNA. In a nutshell, the Monarch Total RNA Miniprep Kit (Cat No.: T2010S, Biolabs Inc.) was used to extract RNA from the caecal tonsils of chick embryos. Following the manufacturer's instructions, the RNA was extracted (*n* = 3 in each group), with a bit of alteration made by applying liquid nitrogen to the sample to crush and homogenize it. The ProtoScript II First Strand cDNA Synthesis Kit (Cat No.: E6560S, Biolabs Inc.) was used to generate cDNA, and the NANODROP ONE (Thermo Scientific, USA) was used to detect the purity and concentration of RNA. The SYBR Green assay Luna Universal qPCR Master Mix (Cat No.: M3003S, Biolabs Inc.) and qTOWER3G Real‐Time PCR (Analytik Jena) were used for the qPCR reaction. The thermal profile involved maintaining the temperature at 95°C for 2 min, denaturing for 5 s, annealing for 30 s at 62°C and extending for 30 s at 72°C. The used primers and gene sequences are as follows—AvBD9: forward primer—AACACCGTCAGGCATCTTCACA, reverse primer—CGTCTTCTTGGCTGTAAGCTGGA, product size—131 bp; IL8L2: forward primer—GGAAGAGAGGTGTGCTTGGA, reverse primer—TAACATGAGGCACCGATGTG, product size—102 bp; IL6: forward primer—AGGACGAGATGTGCAAGAAGTTC, reverse primer—TTGGGCAGGTTGAGGTTGTT, product size—78 bp; housekeeping gene ACTB (β‐actin): forward primer—ATTGTCCACCGCAAATGCTTC, reverse primer—AAATAAAGCCATGCCAATCTCGTC, product size—86 bp (Laptev et al. 2019).

### 2.7 Statistical Analysis

The data analysis in this study was conducted using SPSS version 16, developed by SPSS Inc. Initially, a normality test was applied to the data to assess the distribution of the variables. Following this, the one way analysis of variance (ANOVA) test was used to evaluate the significance of the data. The ANOVA test was used in determining if there are any statistically significant differences among the various group means. The difference was considered significant at the 95% confidence level (*p* < 0.05), highly significant at the 99% confidence level (*p* ≤ 0.01). To further dissect the findings and understand specific group differences, a post hoc analysis using Tukey's Honestly Significant Difference (HSD) test was conducted whenever the ANOVA indicated significant differences.

## 3 Results

### 3.1 Growth Parameters

**FIGURE 1:**
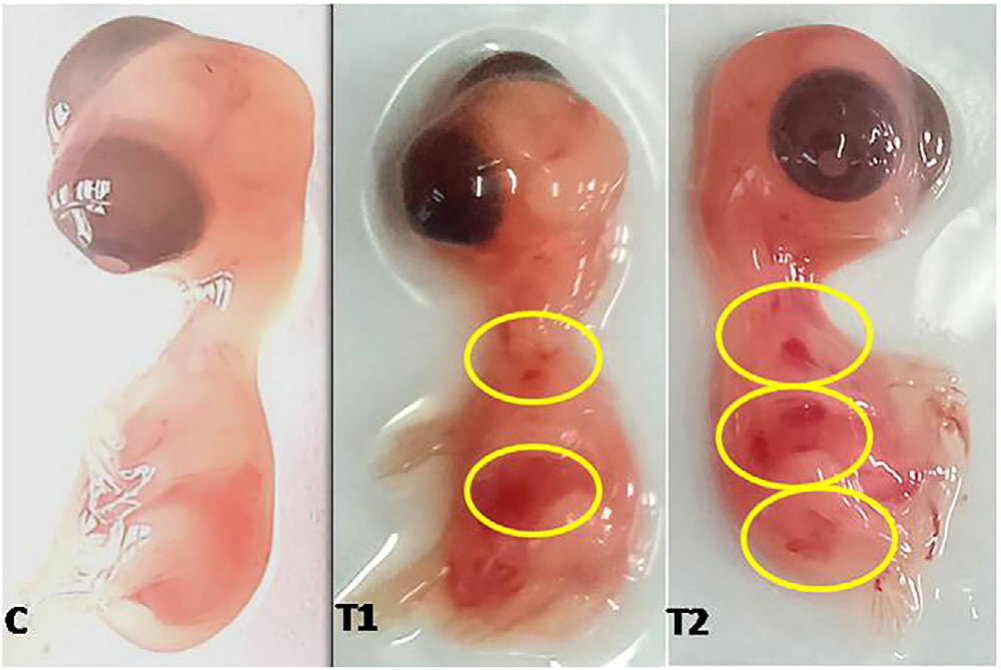
The figure illustrates the 7‐day chick embryos of the TiO_2_ treated group (T1 and T2), displaying subcutaneous haemorrhages marked with circles. In contrast, the control group (C) exhibited no signs of haemorrhage.

**FIGURE 2:**
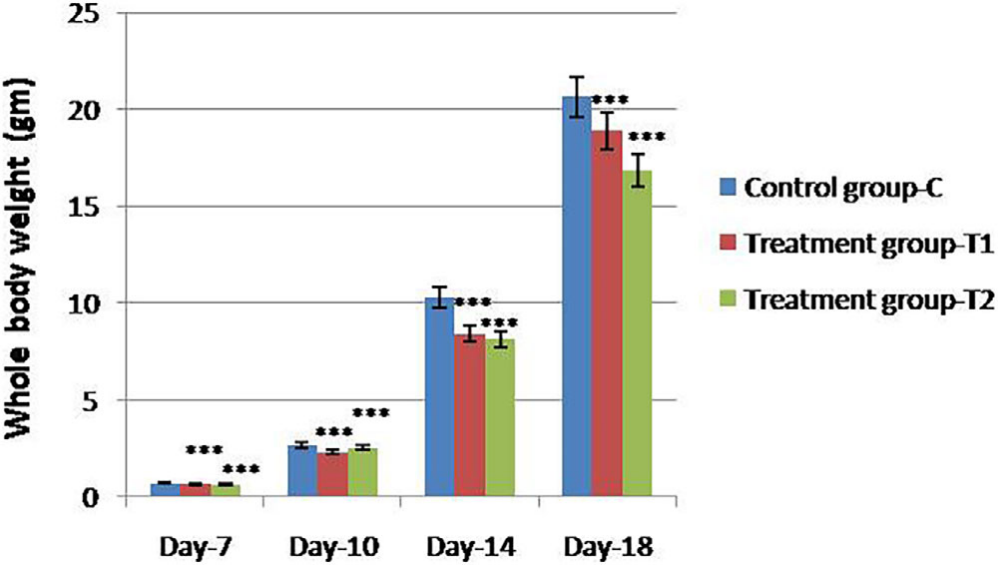
Comparative analysis of whole body weight measures in chick embryos showing control group (C) and titanium dioxide treated groups (T1 and T2). Each group has a sample size of *n* = 5. Statistical analysis reveals a significant difference (***) among the treatment groups versus control group and between treatment groups (*p* < 0.001), ascertaining the impact of TiO_2_ NPs on embryonic development.

In the study, chick embryos at Day 7 treated with TiO_2_ NPs (Groups T1 and T2) showed signs of subcutaneous haemorrhages, as observed in Figure 1. On the other hand, the Group C demonstrated no evidence of such haemorrhages. The exposure of chick embryos to TiO_2_ NPs led to a marked reduction in whole body weight at Days 7, 10, 14 and 18, compared to the control group. Notably, the body weight development in both Groups T1 and T2 was significantly lower when exposed to TiO_2_ NPs, as illustrated in Figure 2. This reduction was statistically significant, with all comparisons between Groups T1 versus T2, T1 versus C and T2 versus C showing notable differences. The means and standard deviation were computed on the basis of a sample size of *n* = 5 for each day group. These variations were statistically significant at a significance level of *p *< 0.01, underscoring the impact of TiO_2_ NP exposure on embryonic development.

### 3.2 Biochemical Parameters of the Amniotic Fluid

Table 1 presents a comprehensive summary of the significant alterations observed in the biochemical parameters of the amniotic fluid of chick embryos following exposure to TiO_2_ NPs. Notably, the levels of urea, creatinine, ALP, AST and ALT demonstrated a statistically significant increase compared to the control group, at a significance level of *p* < 0.05. The study observed dose‐dependent increases in biochemical parameters (urea, creatinine, ALP, AST and ALT) in TiO_2_ NP‐exposed chick embryos, with statistically significant elevations compared to the control group. Significant differences were also observed among the treatment groups for these parameters, indicating a notable impact of the treatments on these biochemical markers.

**TABLE 1:** Biochemical parameters of amniotic fluid in chicken embryos of control and treated groups on Day 18 (*n* = 5).

Biochemical parameters	Control group (C)	Treatment group (T1)	Treatment group (T2)	*p* value
Urea (mg/dL)	8.0604 ± 1.1375	16.1856 ± 1.3118	19.7252 ± 1.511	0.00001^*^
Creatinine (mg/dL)	0.6174 ± 0.0889	0.7358 ± 0.0965	0.77 ± 0.0738	0.0404^*^
ALP (IU/L)	6.6196 ± 1.345	276.4562 ± 4.4053	285.4852 ± 6.6522	0.00001^*^
AST (IU/L)	9.624 ± 0.8441	155.626 ± 2.5301	182.5172 ± 36.028	0.00001^*^
ALT (IU/L)	3.5866 ± 0.5047	50.781 ± 0.9272	56.222 ± 1.5963	0.00001^*^

Abbreviations: ALP, alkaline phosphatase; ALT, alanine aminotransferase; AST, aspartate aminotransferase.

* Significant at *p* < 0.05 (*n* = 5).

### 3.3 Effects on Liver Development

**FIGURE 3:**
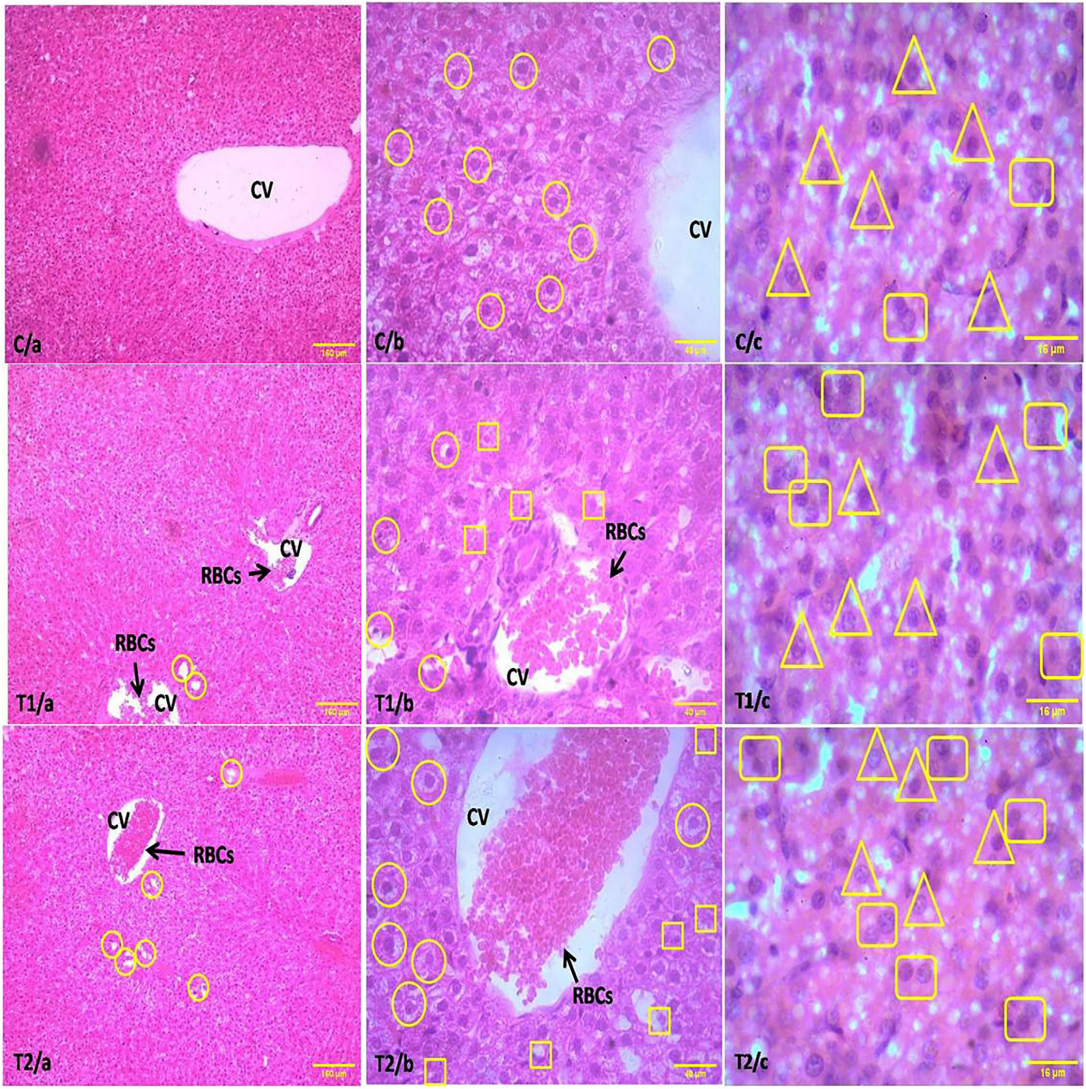
Histological examinations of liver tissues in chick embryos at 18 days of age. C/a, C/b and C/c (10×, 40× and 100× magnification, 160, 40 and 16 µm scale; H&E stain) show the normal liver architecture (C/a and C/b) around the central vein (CV) and highlight single (triangle) and binucleation of hepatocytes (square). In T1/a and T1/b, as well as T2/a and T2/b (10× and 40× magnification, 160 µm and 40 µm scale; H&E stain) depict liver sections from different treatment groups, highlighting variations in CV structure and cellular morphology along with hydropic degeneration (circle) and hepatocytes exhibiting cytoplasmic vacuolation (square). T1/c and T2/c (100× magnification, 16 µm scale; H&E stain) highlight single (triangle) and binucleation of hepatocytes (square). H&E, haematoxylin and eosin.

On the 18th day of the experiment, a detailed examination of liver cross‐sections from the TiO_2_ NPs exposed group revealed a more frequent occurrence of bleeding in the venous canals compared to the control group (Figure 3C/a, C/b). Histological analysis of the liver from the treated groups on Day 18 of incubation (Figure 3T1/a, T2/a) demonstrated a disruption in the typical radial arrangement of hepatocytes around the central vein (CV). Notably, hydropic degenerations were present (indicated by circles), and red blood cells (RBCs) were observed in the CVs. Moreover, at a higher magnification of 40× (Figure 3T1/b, T2/b), the treated groups exhibited hydropic degeneration (indicated by circles) and hepatocytes with cytoplasmic vacuolation (marked by squares). RBCs were observed in the CV of Figure 3T1/b and T2/b. In contrast, liver sections from the control group (H&E stained at 10× and 40× magnifications) (Figure 3C/a, C/b) maintained the radial arrangement of hepatic cords around CV without any noticeable anomalies. In C/c, T1/c and T2/c (100× magnification, 16 µm scale; H&E stain) highlight single (in triangle) and binucleation of hepatocytes (in square). The figures showed a variation in hepatocyte counts, including both uninucleated and binucleated hepatocytes, among the control group (C/c) and the treatment groups (T1/c and T2/c).Table 2 presents a detailed comparative analysis of uninucleated and binucleated hepatocyte counts, as well as the diameter of hepatocytes in liver tissue sections among the control (C) group and two treatment groups (T1 and T2). The results demonstrate a significant variance in the hepatocyte counts and diameters across these groups. In the case of uninucleated hepatocytes, the control group exhibited a count of 38.6 ± 0.5477, which was higher compared to the treatment groups, where T1 recorded a count of 35 ± 0.7071 and T2 showed a count of 33.4 ± 1.1402. This difference was statistically significant, at a significance level of *p* < 0.001. For binucleated hepatocytes, an inverse trend was observed; the control group had a count of 2 ± 0.7071, which was lower than the counts in the treatment groups, with T1 at 5 ± 0.7071 and T2 at 7 ± 0.7071 (*p* < 0.001). Additionally, the diameter of hepatocytes was measured, revealing a mean diameter of 14.86 ± 0.8355 µm in the control group, which was smaller compared to 16.56 ± 0.6229 µm in T1 and 16.86 ± 0.6841 µm in T2, with a significant difference (*p *< 0.001). Significant differences were observed among all the groups for these parameters, indicating a notable impact of the treatments in both the uninucleated and binucleated hepatocyte counts and their diameters.

**TABLE 2:** Uninucleated and binucleated hepatocytes count in liver tissue section comparing control (C) and treated (T1 and T2) groups.

Parameters	Control (C)	Treatment (T1)	Treatment (T2)	*p* value
Uninucleated hepatocytes	38.6 ± 0.5477	35 ± 0.7071	33.4 ± 1.1402	0.00001^*^
Binucleated hepatocytes	2 ± 0.7071	5 ± 0.7071	7 ± 0.7071	0.00001^*^
Diameter of hepatocyte (µm)	14.86 ± 0.8355	16.56 ± 0.6229	16.86 ± 0.6841	0.001784^*^

* Significant at *p* < 0.001 (*n* = 5).

### 3.4 Effects on the Development of Caecal Tonsil

**FIGURE 4:**
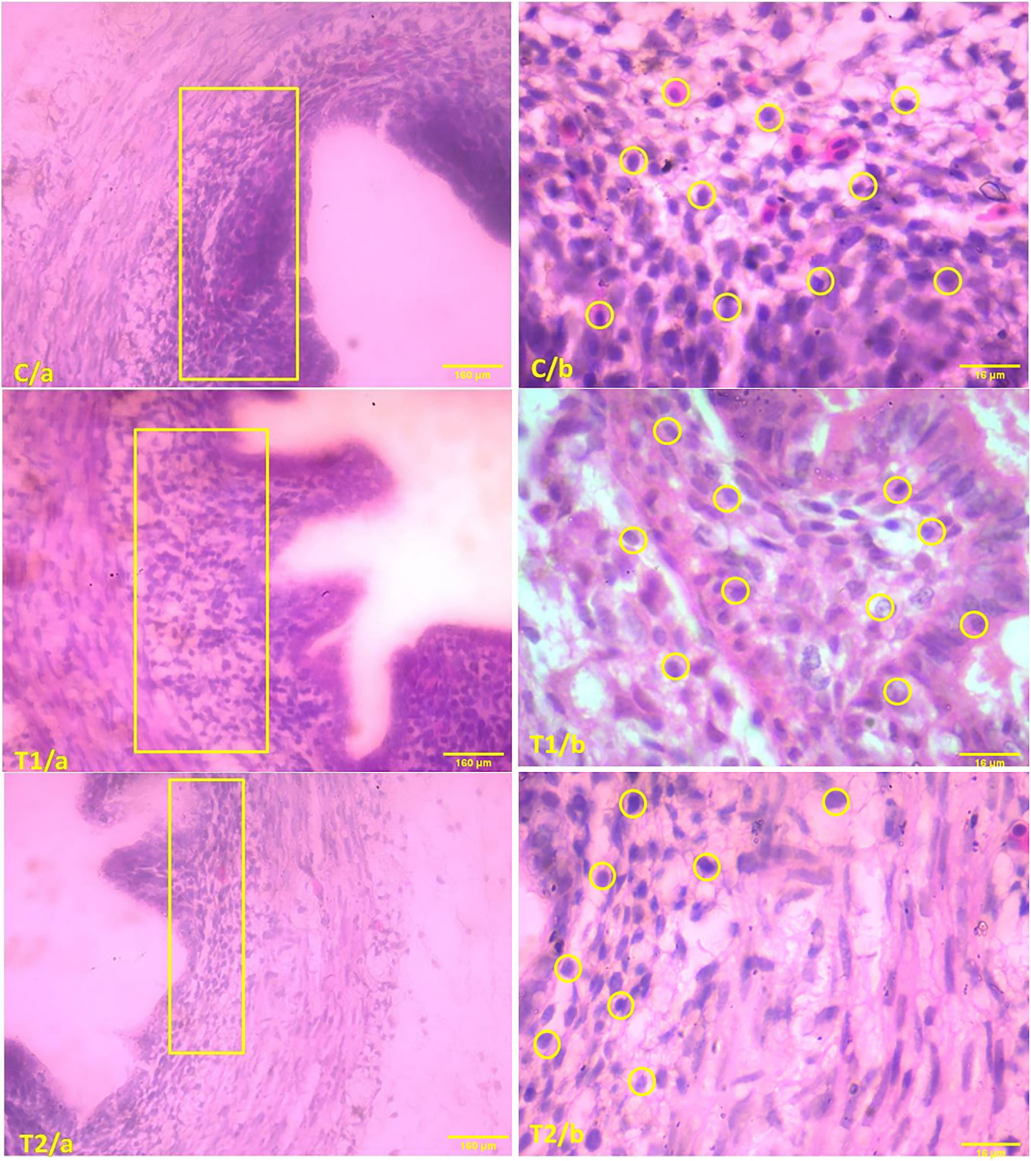
Histological comparison of caecal tonsils in 18‐day chick embryos. Images show sections stained with H&E at varying magnifications (10×, 160 µm; 100×, 16 µm). In C/a and C/b represent the control group, T1/a and T1/b represent the group treated with 150 µg/mL, and T2/a and T2/b show the group treated with 300 µg/mL. Lymphocyte density in the lamina propria is highlighted using rectangles and circles.

Figure 4C/a, displaying an 18‐day‐old chick embryo from the control group under 10× magnification (H&E stain), reveals a dense accumulation of lymphocytes (scale bar: 160 µm) within the lamina propria. This observation is further supported by Figure C/b, which, at 100× magnification (H&E stain, scale bar: 16 µm), confirms the high lymphocyte density in the control group. In contrast, Figure 4T1/a (10× magnification, H&E stain, scale bar: 160 µm) shows a reduction in lymphocyte count by 150 µg/mL in the treatment groups. Similarly, Figure T1/b (100× magnification, H&E stain, scale bar: 16 µm) shows a decreased lymphocyte density in the lamina propria of the treatment group. This reduction is even more evident in Figure T2/a (10× magnification, H&E stain, scale bar: 160 µm), where the lymphocyte density is significantly lower (300 µg/mL) compared to both the T1 and control groups. Consistent with this, Figure T2/b (100× magnification, H&E stain, scale bar: 16 µm) demonstrates that the lamina propria in the 300 µg/mL treatment group contains fewer lymphocytes than those in the T1 and control groups.

Table 3 presents a numerical analysis of lymphocytes in the caecal tonsils from both control and treated groups. The data indicate that the total lymphocyte count in the control group (C) was significantly higher (240 ± 13.51) compared to the treated groups (T1 and T2), which recorded counts of 205.8 ± 9.28 and 165 ± 7.65, respectively (*p* < 0.05). Significant differences were observed among the treatment groups for these parameters, indicating a notable impact of the treatments on the number of lymphocyte.

**TABLE 3:** Lymphocyte count in caecal tonsil comparing control (C) and treated (T1 and T2) groups.

Group	Control (C)	Treatment group (T1)	Treatment group (T2)	*p* value
Number of lymphocytes	240 ± 13.51	205.8 ± 9.28	165 ± 7.65	0.00001^*^

* Significant at *p* < 0.001 (*n* = 5).

### 3.5 Effects on the Development of Bursa of Fabricius

**FIGURE 5:**
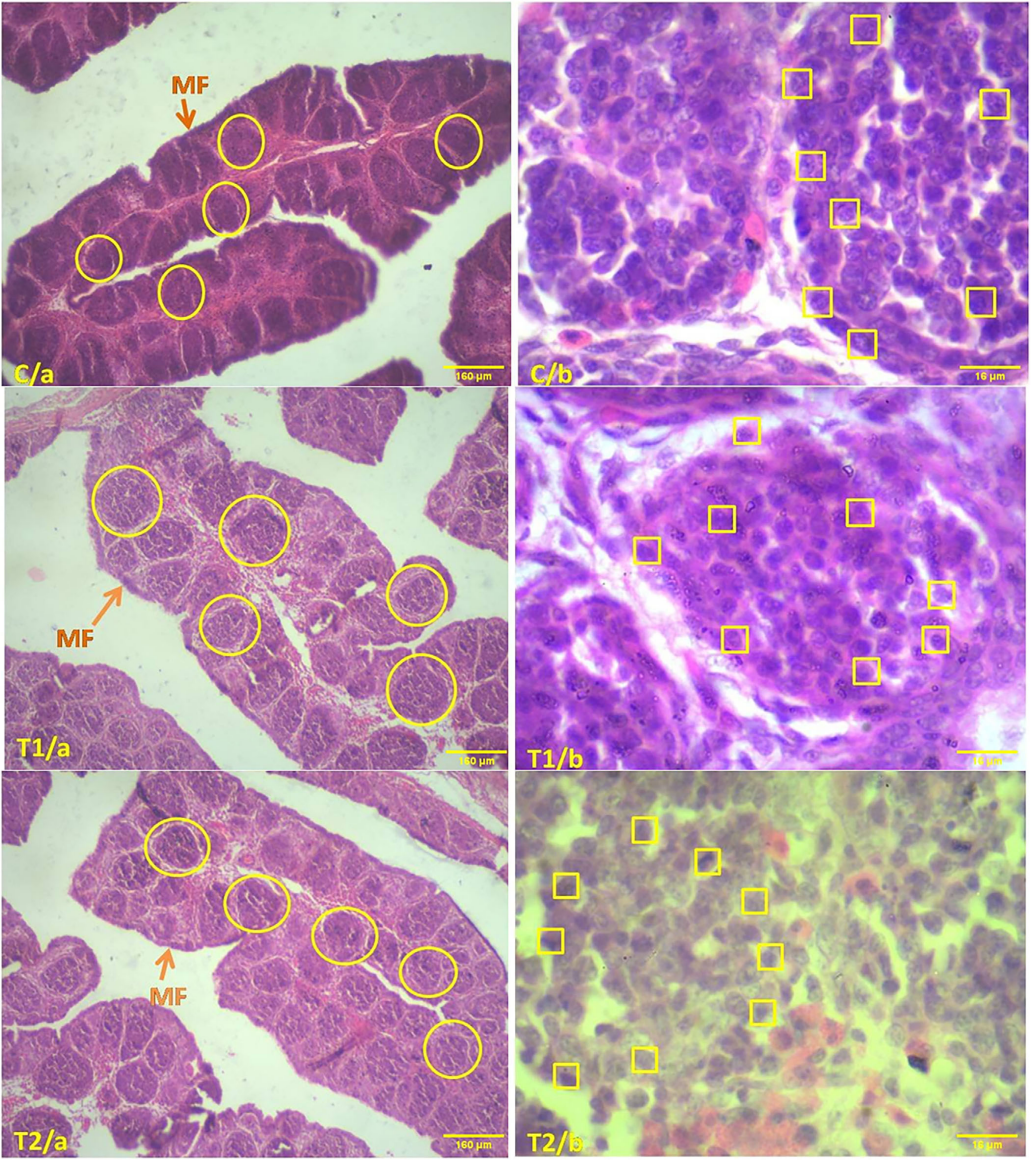
Comparative histological examination of the bursa of Fabricius in 18‐day chick embryos. Figure C displays the control group's lymphatic follicles (C/a: H&E stain, 10×, 160 µm; C/b: H&E stain, 100×, 16 µm). Figure T1 illustrates the group treated with 150 µg/mL TiO_2_ (T1/a: H&E stain, 10×, 160 µm; T1/b: H&E stain, 100×, 16 µm). Figure T2 shows the group treated with 300 µg/mL TiO_2_ (T2/a: H&E stain, 10×, 160 µm; T2/b: H&E stain, 100×, 16 µm). Each image highlights the density of lymphocytes in the lymphatic follicles using square and circles. H&E, haematoxylin and eosin.

In this histological study of the bursa of Fabricius in 18‐day‐old chick embryos, histological examination revealed that the average number of mucosal folds (MF) remained consistently at 12 in all examined groups. Detailed analysis of the follicles within each fold showed no significant differences, as present in the accompanying table (Table 4). Representative micrographs from the control group (Figure 5C/a and C/b), stained with H&E and observed at 10× (160 µm) and 100× (16 µm) magnifications, respectively, demonstrate a pronounced aggregation of lymphocytes within the lymphatic follicles, as indicated by the circled areas. Closer examination reveals square markers denoting increased lymphocyte presence within these follicles. Conversely, the group treated with a 150 µg/mL concentration (Figure 5T1/a, stained with H&E, 10× magnification, 160 µm scale bar) exhibited a reduced density of lymphocytes within the lymphatic follicles compared to the control group, as highlighted by circular markers. This trend is also observed at higher magnification (Figure 5T1/b, stained with H&E, 100×, 16 µm), revealing a significant reduction in lymphocyte density within the lymphatic follicles compared to the control.

**TABLE 4:** Mucosal fold (MF) count in bursa of Fabricius comparing control (C) and treated (T1 and T2) groups.

Group	Control (C)	Treatment group (T1)	Treatment group (T2)	*p* value
Number of mucosal fold (MF) per bursa of Fabricius	12.2 ± 0.84	12 ± 0.71	11.8 ± 0.84	0.735092
Number of lymphatic follicles per MF	83.6 ± 4.22	81.2 ± 3.12	73.4 ± 2.70	0.001259^*^
Number of lymphocytes per slide	255.8 ± 7.43	249.8 ± 4.15	221.2 ± 13.00	0.000115^*^

* Significant at *p* < 0.05 (*n* = 5).

Further detailed analysis of the group treated with a 300 µg/mL concentration (T2/a) and stained with H&E at 10× magnification reveals a significant reduction in lymphocyte density within the lymphatic follicles, as denoted by circular markers. This observation is supported by high‐magnification micrographs (T2/b, stained with H&E, 100×, 16 µm), indicating that the 300 µg/mL treatment group has fewer lymphocytes in the lymphatic follicles compared to both the T1 group and the control.

Statistical analysis shows no significant difference in the number of MF between the control group (12.2 ± 0.84) and the two treatment groups (12 ± 0.71, 11.8 ± 0.84) as presented in Table 4. However, a statistically significant difference in the number of lymphatic follicles was observed among the groups, with the control group exhibiting the highest count (83.6 ± 4.219) and treatment group T2 the lowest (73.4 ± 2.7019). Additionally, a significant variance in lymphocyte counts was noted, with the control group having the highest count (255.8 ± 7.4297) and treatment group T2 the lowest (221.2 ± 13.0077), as detailed in Table 4. It was revealed dose‐dependent effects in the bursa of Fabricius, with variations in lymphocyte density and lymphatic follicle counts. Although the mean number of MF remained constant, significant variations in lymphocyte counts across treatment groups were noted, indicating a dose‐dependent impact.

### 3.6 Immunity Genes Expression

**FIGURE 6:**
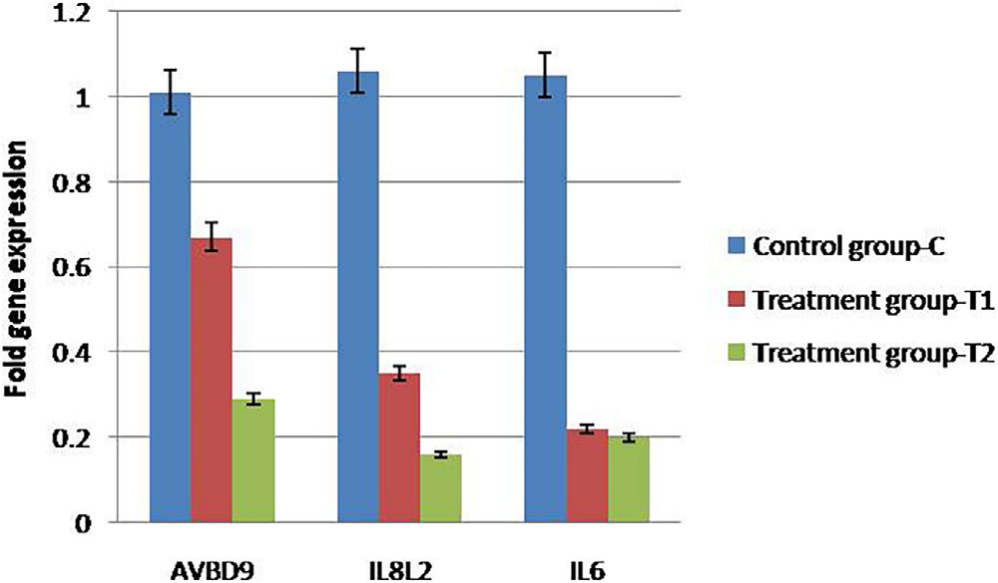
This figure depicts the expression levels of immunogenic genes *AvBD9, IL8L2* and *IL6* in the chicken embryo following treatment with Titanium dioxide. In experimental groups T1 and T2, a notable decrease in the expression of these genes is observed compared to the control group. The sample size for each group is *n* = 3. Statistical analysis (ANOVA, *p* < 0.05) confirms the significant downregulation of these genes due to TiO_2_ NPs exposure, underlining its potential immunomodulatory effects. ANOVA, one way analysis of variance; TiO_2_ NPs, titanium dioxide nanoparticles.

On the 18th day of the incubation period, a qPCR test was performed to assess the expression levels of the immunity genes *AvBD9, IL8L2* and *IL6*. Compared to the control group (C), the treated groups (T1 and T2) exhibited a reduction in the mRNA expression of these immunity genes. The expression of immunity genes (*AvBD9, IL8L2* and *IL6*) was also decreased in a dose‐dependent manner in treated groups compared to the control group, as evidenced by qPCR analysis on Day 18 of incubation (Figure 6).

## 4 Discussion

In the course of this investigation, spanning from Days 1 to 18, monitoring was done meticulously both control and treated groups for adverse effects. It was observed that there were no fatalities or negative consequences attributable to the incubation conditions in either group. This study revealed no significant abnormalities except for subcutaneous bleeding under the skin.

Significantly, the treated group exhibited a notable decrease in overall body weight when compared to the control group. This study specifically focuses on the impact of TiO_2_ NPs on the growth of chicken embryos. A key finding was the evident reduction in body weight among the treated group. This observation is consistent with the findings of Duan et al. (2010) in mice, who reported a similar pattern of weight loss in mice exposed to higher doses of TiO_2_ NPs. Such parallel outcomes across different species suggest a potential universal biological mechanism, where exposure to TiO_2_ NPs adversely affects normal weight gain. However, this study's findings stand in contrast to other research, namely those by Wang et al. (2007); Liu et al. (2009) and Ma et al. (2009), which did not observe significant weight changes in mice following nanoparticle exposure. These divergent results underscore the complexity inherent in the interaction of nanoparticles within biological systems. Factors such as varying experimental methodologies, differences in exposure durations and species‐specific responses may contribute to this inconsistency. The concept of species‐specific susceptibility provides a plausible explanation for these divergent results. Different species possess unique physiological and genetic profiles, leading to varied responses to similar treatments. Factors, like metabolic rates, absorption efficiency and cellular response mechanisms, differ significantly across species (Burton et al. 2011; Van den Berg et al. 2019). These inherent biological differences may account for the observed variations in response to TiO_2_ NP exposure. Consequently, although the findings of the present study contribute valuable insights into the effects of TiO_2_ NPs, they should be contextualized within the specific species studied. Extrapolating these results to other species may not be straightforward due to these interspecies variations. Furthermore, one possible mechanism is the impact of TiO_2_ NPs on cellular and molecular pathways that regulate growth and metabolism. These nanoparticles may interfere with nutrient absorption or metabolism, leading to reduced growth rates. Additionally, TiO_2_ NPs might induce oxidative stress or inflammation, which can negatively affect overall physiological health and growth (Rinninella et al. 2021; Gojznikar et al. 2022).

The current study supports prior findings, revealing that exposure to TiO_2_ NPs significantly alters the biochemical parameters in the amniotic fluid of chicken embryos. This alteration is evidenced by marked increases in the concentrations of urea, creatinine, ALP, AST and ALT following TiO_2_ NPs treatment. These findings are consistent with those reported by Duan et al. (2010) in mice, where TiO_2_ NPs were observed to elevate levels of ALT, ALP and AST. The observed disturbances in metabolic processes and oxidative stress, particularly in liver and kidney tissues, likely contribute to the changes in these biochemical markers. This is evidenced by the elevated levels of these markers. Research indicates that exposure to TiO_2_ NPs can lead to hepatotoxicity and nephrotoxicity in developing organisms, as suggested by Shukla et al. (2014) and Zhang et al. (2023). Given the crucial roles of the liver and kidneys in maintaining biochemical equilibrium and overall health, the significant alterations in these biochemical parameters imply that TiO_2_ NPs could adversely affect the physiological development of chicken embryos, as noted by Shabbir et al. (2021). Furthermore, the potency of these biochemical markers increases with growth, indicating that TiO_2_ NPs exposure leads to cellular damage and metabolic alterations. These observations underscore the importance of understanding the mechanisms driving changes in biochemical parameters and their implications for embryonic health and well‐being. Additionally, the research demonstrates that TiO_2_ NPs treatment modifies the morphology and histology of liver tissue in chicken embryos. This aligns with findings from previous studies conducted on mice by Duan et al. (2010), Wang et al. (2007) and Liu et al. 2009; Liu et al. 2011), which also reported alterations in liver structure and function following TiO_2_ NPs exposure. Wang et al. (2007)) observed in mice that TiO_2_ nanoparticulate suspensions increased hepatic coefficients, CV damage and patchy hepatocyte necrosis. Similarly, the research by Liu et al. (2009) observed that exposure to TiO_2_ NPs in mice led to increase liver coefficients and compromised liver functionality. Additionally, this exposure activated inflammatory responses and caused histological alterations in liver tissue. These findings suggest that nanoparticulate TiO_2_ may adversely affect both immune responses and liver health, potentially resulting in liver damage. The histological examination and comparative analysis of hepatocytes in liver tissue, underscores the potential influence of the treatments on hepatocyte proliferation or stability. These findings suggest that the treatments may promote binucleation, a phenomenon often associated with cell stress or regeneration processes. Additionally, the diameter of hepatocytes varied significantly among the groups, indicating a possible impact of the treatments on hepatocyte morphology. This aligns with existing literature that associates such cellular alterations with various physiological and pathological conditions, reflecting the dynamic nature of liver tissue in response to external stimuli (Duan et al. 2010; Wang et al. 2007). Collectively, the present findings and previous studies suggest that TiO_2_ NPs may exhibit hepatotoxic properties, particularly at higher dosages. This highlights the necessity for a thorough assessment of the developmental and physiological impacts of TiO_2_ NPs. Understanding the molecular aetiology and cellular mechanisms underlying TiO_2_ NP‐induced liver damage is crucial for guiding regulatory and safety measures regarding the use of TiO_2_ NPs in various applications.

This research investigated the effects of TiO_2_ NPs on the caecal tonsil and the bursa of Fabricius in chicken embryos. The results revealed notable alterations in the counts of lymphocytes and lymphatic follicles, highlighting the significant effects of TiO_2_ NPs exposure on the immune and developmental systems in chicken embryos. The treatment groups, T1 and T2, exhibited a significantly reduced lymphocyte count in the lamina propria of the caecal tonsil compared to the control group. This finding aligns with the research conducted by Duan et al. (2010), which demonstrated that nanoparticulate TiO_2_ diminishes the expression of immunity genes in mice. Notably, their study underscored that the introduction of nanoparticulate TiO_2_ led to a marked decrease in *interleukin‐2* (*IL‐2*) protein levels. This reduction was dose‐dependent, suggesting a potential compromise in the immunological response. This correlation between TiO_2_ exposure and impaired immune function provides a critical insight into the effects of nanoparticles on biological systems. Bettini et al. (2017) observed in rat that the substantial decrease in the CD4/CD8 ratio in their study indicated an increase in T cell proliferation accompanied by a diminished functional capacity of these cells, adversely impacting the immune system. The findings suggest that TiO_2_ NPs might influence the development of lymphocyte through immunomodulatory mechanisms. They also found that TiO_2_ NPs adversely affect both intestinal and systemic immune responses. Furthermore, the present research focusing on the bursa of Fabricius revealed a statistically significant variation in lymphatic follicle and lymphocyte counts between the control group and those subjected to treatment. Notably, the highest counts were observed in the control group, whereas the treatment group T2 exhibited the lowest figures. This variation implies that TiO_2_ NPs could disrupt the lymphocyte populations in the bursa of Fabricius, potentially altering the immune response and its functionality.

The present study revealed that the experimental groups exhibited a reduced expression of *AvBD9, IL8L2* and *IL6* mRNA compared to control groups, highlighting a significant immunomodulatory effect of TiO_2_ NPs on avian species. This finding aligns with the previous research on different species, but our study uniquely demonstrates these effects during the embryonic development stage in birds.

Park et al. (2008) explored the effects of TiO_2_ NPs on BEAS‐2B, a human bronchial epithelial cell line, concluding that TiO_2_ NPs potentially disrupt calcium regulation in lymphocytes, leading to oxidative stress and cellular damage. This disturbance is believed to suppress immune‐related gene expression, suggesting the immunomodulatory influence of TiO_2_ NPs. Similarly, Tuncsoy and Mese (2021) found that TiO_2_ NPs reduced haemocyte counts in insects due to oxidative damage. Sang et al. (2012) reported a decrease in lymphocyte counts in mice exposed to TiO_2_ NPs for 90 days, highlighting the cytotoxic effects on lymphocyte populations and immune gene expression. Zhao et al. (2021) suggested that decreased immune responses might be associated with liver tissue disturbance and impaired function. In contrast, some studies report differing effects of TiO_2_ NP exposure. For instance, Fu et al. (2014) observed an increase in T‐ and B‐cell production in Sprague Dawley rats following direct lung exposure to TiO_2_ NPs, and Wang et al. (2007) noted a rise in T cells in the spleen of C57BL/6 mice due to nanoparticle exposure. These variations may be attributed to differences in particle size, shape, delivery method, dose, exposure duration, crystal structure, species‐specific responses and surface charge (Ayorinde and Sayes 2023).

Our study provides novel insights by focusing on the embryonic stage of avian species. Unlike previous studies, we evaluated the effects of TiO_2_ NPs through avian diets, considering their unique dietary habits and body weight. This approach allowed us to understand the ecological and health implications of TiO_2_ NPs on birds more comprehensively. Additionally, we examined changes in the embryonic microenvironment, including biochemical alterations in amniotic fluid and the regulation of immunity genes due to nano‐TiO_2_ exposure. This focus on the embryonic stage offers new perspectives on the developmental and physiological effects of TiO_2_ NPs on avian species, contributing to the broader understanding of nanoparticle exposure's impact on immune function and development across different biological systems.

The present study showed that TiO_2_ NPs reduced the expression of immunity genes. This alteration may be due to TiO_2_ NPs, inducing oxidative stress, leading to DNA damage, protein oxidation and lipid peroxidation (Caruso et al. 2023). This stress is initiated through the generation of ROS, disrupting cellular functions (Trouiller et al. 2009; Gojznikar et al. 2022). TiO_2_ nanoparticles also interact with cellular components, potentially causing apoptosis or necrosis (Cao et al. 2023). Furthermore, they trigger inflammatory responses, including cytokine production and macrophage activation, leading to tissue damage. Additionally, these nanoparticles influence cell signalling pathways, affecting cell proliferation, differentiation and survival. The genotoxic effects, such as chromosomal aberrations and mutations, further elucidate the comprehensive impact of TiO_2_ nanoparticles on biological systems (Zhao et al. 2009; Dong et al. 2023).

The current study investigates the impact of TiO_2_ NPs on avian species, with a particular emphasis on embryonic development. Building upon foundational research by Weir et al. (2012), this study evaluates the effects of TiO_2_ NPs exposure through avian diets, taking into account their unique dietary habits and body weight considerations. This approach aids in understanding the ecological and health implications of TiO_2_ NPs on birds. Additionally, the research explores changes in the embryonic microenvironment, examining biochemical alterations in amniotic fluid and the regulation of immunity genes due to nano‐TiO_2_. This focus on the embryonic stage offers new insights into the developmental and physiological effects of nano‐TiO_2_ on avian species.

## 5 Conclusion

In summary, our study reveals that TiO_2_ NPs significantly impact the immunomodulatory, hepatic and metabolic processes in chicken embryos. Notably, TiO_2_ NPs treatment led to a marked reduction in the expression of key immunity genes (*AvBD9, IL8L2* and *IL6*) relative to the control group, indicating potential immunomodulatory effects. Furthermore, a reduction in body weight and subcutaneous haemorrhages in the treated group was observed, which points to the influence of TiO_2_ NPs on the physiological development of chicken embryos. Alarmingly, there was a significant increase in the levels of urea, creatinine, ALP, AST and ALT in the amniotic fluid, suggesting potential hepatotoxic and nephrotoxic effects on the developing embryos. Histological examinations also revealed TiO_2_ NP‐induced alterations in liver tissue and immunological changes in the bursa of Fabricius and caecal tonsil. This research emphasizes the critical importance of closely monitoring exposure to TiO_2_ NPs, particularly in light of their increasing prevalence across multiple industries. This vigilance is essential for understanding and mitigating the ecological and health impacts of TiO_2_ NPs on bird populations, considering their specific dietary habits and body weight dynamics. Importantly, the results offer essential insights into the broader environmental and human health implications of nanoparticles, extending beyond the scope of chick embryos. In light of the growing interest in the neurodevelopmental and neurodegenerative impacts of nanoparticles, future investigations should explore the potential effects of TiO_2_ NPs on neurological development and degeneration.

## Author Contributions

**Md. Sadequl Islam**: conceptualization, data curation. methodology, investigation, supervision, writing–original draft, writing–review and editing. **Md. Nurul Amin**: conceptualization, methodology, writing–review and editing. **Mst. Deloara Begum**: conceptualization, investigation, writing–review and editing.

## Ethical Statement

The Ethical Committee of the Institute of Research and Training (IRT) at Hajee Mohammad Danesh Science and Technology University has authorized the approach for preserving chick embryos in laboratory research and collecting samples. The reference number for this approval is HSTU/IRT/4209.

## Conflicts of Interest

The authors declare no conflicts of interest.

### Peer Review

The peer review history for this article is available at https://www.webofscience.com/api/gateway/wos/peer-review/10.1002/vms3.70105

## Data Availability

The corresponding author will provide all data supporting the research upon request.
